# An inside guide to eLife digests

**DOI:** 10.7554/eLife.25410

**Published:** 2017-03-15

**Authors:** Stuart RF King, Emma Pewsey, Sarah Shailes

**Affiliations:** *eLife*, Cambridge, United Kingdom; *eLife*, Cambridge, United Kingdom; *eLife*, Cambridge, United Kingdom

**Keywords:** plain-language summaries, scientific publishing, public engagement

## Abstract

After summarizing over 2,400 articles in plain language, the eLife Features team shares what it has learnt about writing and editing for a broad audience.

Nearly every profession or trade has its own technical language or jargon, and science is no exception. Scientists in every discipline rely on a specific vocabulary of well-defined words and phrases to communicate efficiently with their peers. In fact, this vocabulary, combined with a formal way of writing, means that precise statements can be made in only a few words. Yet this kind of economy also comes at a cost; it can prevent people from outside of the field understanding what is written or said.

Consider the word "trogocytosis", for example. Put simply, those 12 letters describe a process whereby certain types of white blood cell briefly fuse with another cell so that some proteins are transferred between the membranes of the two cells. However, even if you know that "*trogo*" is essentially Greek for gnaw or nibble, and that "*cytosis*" refers to the transport of molecules into or out of cells, the exact meaning of the word is still far from obvious. You either know its meaning or you don’t.

Plain-language summaries are one way to combat this exact issue, and eLife has included such summaries – called eLife digests – in research articles since it first started publishing in 2012. An eLife digest is intended to briefly explain the background and significance of a research paper in words that people outside the field can understand. Readers of the digests range from scientists working across the life sciences to interested individuals who do not have a science degree.

Over 2,400 eLife digests have been published to date. With an average length of 347 words, that number brings the total word count to over 832,800. That is equivalent to over four copies of *On the Origin of Species*, or almost one-and-half-times the length of *War and Peace*. It would also get someone two thirds of the way into the sixth Harry Potter book – *Harry Potter and the Half Blood Prince* – if they read from the beginning of the series.

**Figure 1:**
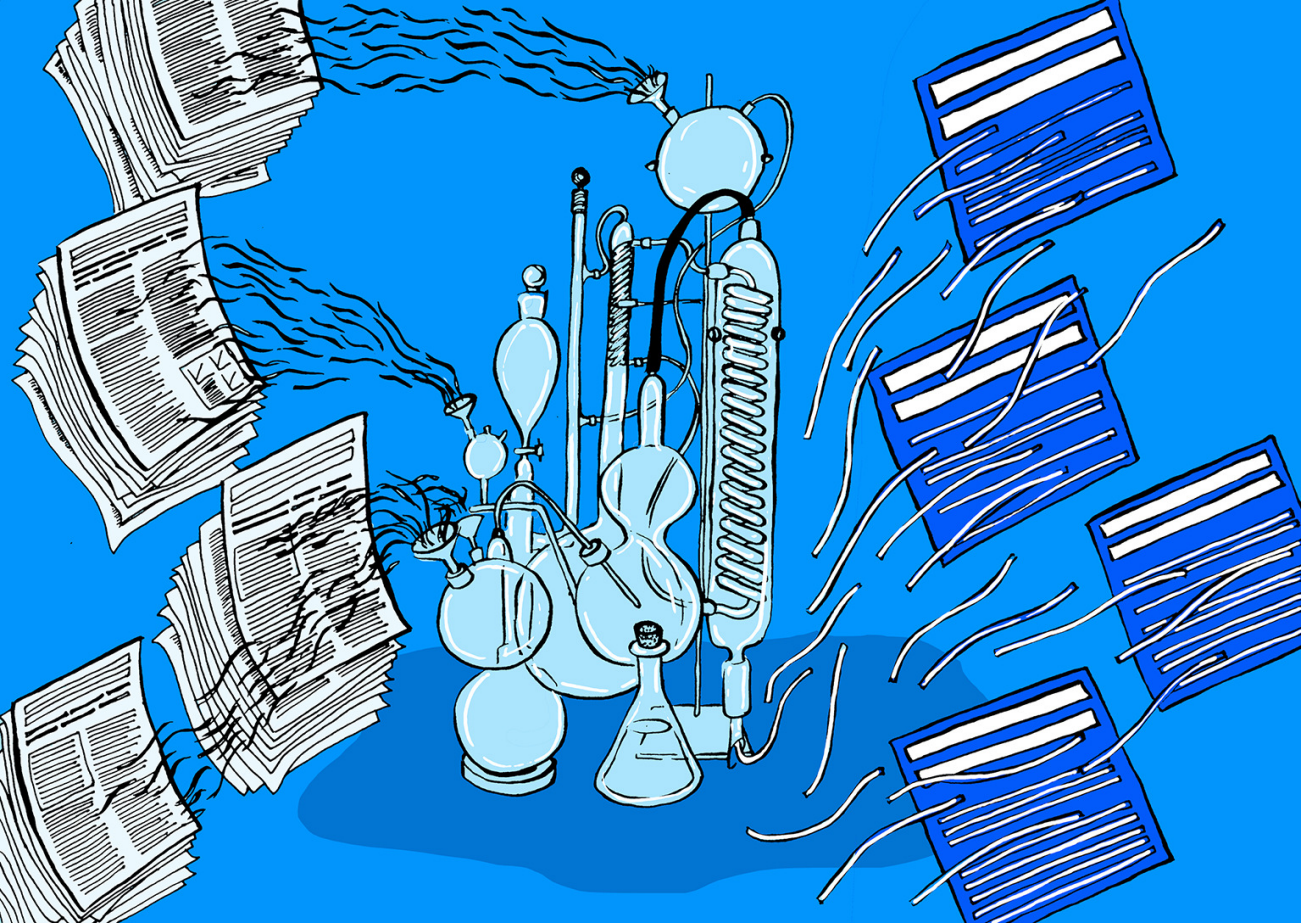
eLife digests distil the background and significance of scientific papers into language that is accessible to non-scientists too.

IMAGE CREDIT: vividbiology.com.

## Motivation

So, what is the point of eLife digests? Firstly, we see digests as part of a wider effort to make original research as open and accessible as possible. Being an open-access journal means that anyone (with an Internet connection) can freely read articles published in eLife. The digests should mean that the majority of those readers can also learn something about the latest research results reported in the journal, regardless of their background.

Secondly, eLife is a journal with a broad scope and eLife digests are one small way that we can help to foster interdisciplinary research. A plant biologist with decades of experience in research, for example, is unlikely to also be an expert in neuroscience (and vice versa). By explaining the findings of a paper in plain language, we hope that digests will help other scientists to identify new connections between different scientific disciplines.

Lastly, from giving talks to writing papers, communication is part of the everyday life of a scientist. However, not every scientist gets formal training in communication skills. By working on an eLife digest, the authors of the original research article gain skills and experience that can be applied to other situations.

## The process explained

So, who writes an eLife digest? Being aimed at a more general audience, the digests have always been handled by the Features editors who also look after Insights, interviews, podcasts and the other magazine-style content in the journal. Together with some freelance writers, we write most eLife digests. Every digest is edited carefully and sent to the authors of the research paper for checking before it is published; we work closely with the authors to ensure that each digest is a clear and accurate explanation of the main findings of their paper.

Writing an eLife digest for a paper is definitely not "dumbing it down". That rather arrogant phrase (which thankfully we do not hear too often) shows disrespect to those interested readers who, either because of a lack of opportunity or inclination, just so happen not to be experts in the field. Instead the challenge is to explain the same concepts and findings as the paper but in language that most people would understand, and without assuming that the reader has any previous specialist knowledge.

The writing style in a digest should also be more active and engaging than the passive and formal style that is characteristic of most scientific articles. However, this does not mean that digests should be viewed as exercises in creative writing either: flowery language and convoluted analogies can be just as difficult to follow as text laden with scientific jargon. Instead, a good eLife digest is clear, concise and complete; it states the facts without resorting to exaggeration or hype.

Each eLife digest is published in a prominent position, immediately below the abstract of its research article. However, the intended audience also includes people who do not normally visit journal websites. To reach more of these readers, we regularly re-publish some digests on Medium, a social publishing platform.

## Lessons learned

After almost five years of publishing eLife digests, what have we learnt about writing plain-language summaries? We are confident that you can write a digest for any research paper, including papers that would not typically attract the attention of the wider media. Sure, we have written digests about the discovery of *Homo naledi* and how one in four shark species are on the brink of extinction. But we have also tackled topics like how scuttle flies develop as embryos, how proteins form changing patterns inside artificial chambers, and how sodium and potassium ions are pumped across cell membranes. We feel that digests are especially valuable in such papers because they are unlikely to be highlighted in a press release or attract coverage in the media.

Over the years, we’ve also experimented with different approaches to preparing digests and refined our process. We have found, for example, that providing regular feedback to freelance writers results in better drafts that require less editing and revision.

We have also found that most authors are willing to get involved in preparing an eLife digest for their paper if given the opportunity. Initially, the digests were written using the accepted manuscript and authors’ cover letter as the primary sources of information. However, reading this material, getting familiar with the background and then distilling it down to the key findings took a lot of time. In 2015, we started to think about how we could involve the authors of the paper – who already knew all of these details – earlier in the process. From May to July 2015, we ran a pilot study with 100 authors who had recently been invited to submit a revised manuscript. We asked these authors about the key points that should be covered in the digest, and gave some brief guidelines on how we would like them to answer our questions.

This pilot study worked better than we had expected; most authors sent us some answers (79 out of 100), often before their manuscript had even been accepted. Importantly, most of the answers were useful. Rather than starting from scratch, we could edit them for clarity and style, and fill in any gaps by reading the accepted manuscript. Authors also made fewer edits than before when checking their digests, and now we ask authors to answer four questions to help us write their digest. (More details on the preparation of eLife digests are available in this blogpost).

We also learnt that most authors underestimate how much they need to change their writing style to write for a non-specialist audience. This was not unexpected because we appreciate that being immersed in their research can make it harder for authors to write for people who do not know what they know, the so-called curse of knowledge. Nevertheless, giving clear instructions and breaking down the task by asking authors a few questions has, in our experience, been a good way to tap into their expert knowledge and write the digest more quickly.

Refining the process helped us to prepare eLife digests for every paper even as the journal grew. By 2016, however, it was clear that writing a digest cannot be rushed. As the number of papers accepted each month continued to climb, we made the difficult decision to stop publishing a digest for every research article. Instead, we now identify a selection of about 60 papers each month for eLife digests. These are selected based on a range of criteria: first and foremost, we want to offer digests to those authors who show the most willing to get involved early on in the process. Secondly, we rely on recommendations from eLife’s Board of Reviewing Editors to find those papers that are the most significant within their fields. Lastly, we select other papers from areas that most interest our readers, and especially those papers that attempt to bridge different disciplines.

## Progress so far

So are the digests "working" as intended? That’s a difficult question to answer and one that we’ve only recently started looking into seriously. What we do know is that well over 90% of authors tell us that they’d like to work with us to prepare an eLife digest for their paper. We also know that when people read research articles on the eLife website each month, somewhere between 13% and 25% of the time they read the digest.

Based on surveys, we know that the current audience is mainly scientists, though some non-scientists regularly read digests too. Among the scientists, there was a slight preference for reading digests in papers in their own field as opposed to other fields of research, but 93% of those who read digests in other fields found them useful. Lastly, scientists and non-scientists alike felt that more journals should consider providing plain-language summaries of research articles. (More details about a survey of digest readers are available in this blogpost).

In the future, we’ll be looking to ask authors about their motivations, expectations and experience of writing plain-language summaries. In particular, we’d like to get a better understanding of what they might gain from working on an eLife digest for their paper, and if we can help them more. In addition, we will be increasing our efforts to reach new audiences, and make digests more discoverable in general. Finally, we hope that, by sharing our experiences, we might encourage others – from publishers to individual scientists – to join us in taking steps to make more scientific research not just open access but also as accessible as possible.

